# Assessing global and regional public interest in home dialysis modalities from 2004 to 2024

**DOI:** 10.3389/fneph.2024.1489180

**Published:** 2024-11-13

**Authors:** Charat Thongprayoon, Wannasit Wathanavasin, Supawadee Suppadungsuk, Paul W. Davis, Jing Miao, Michael A. Mao, Iasmina M. Craici, Fawad Qureshi, Wisit Cheungpasitporn

**Affiliations:** ^1^ Division of Nephrology and Hypertension, Department of Medicine, Mayo Clinic, Rochester, MN, United States; ^2^ Nephrology Unit, Department of Medicine, Charoenkrung Pracharak Hospital, Bangkok Metropolitan Administration, Bangkok, Thailand; ^3^ Chakri Naruebodindra Medical Institute, Faculty of Medicine Ramathibodi Hospital, Mahidol University, Samut Prakan, Thailand; ^4^ Division of Nephrology and Hypertension, Department of Medicine, Mayo Clinic, Jacksonville, FL, United States

**Keywords:** peritoneal dialysis, home hemodialysis, home dialysis, renal replacement therapy, public interest, geographic distribution

## Abstract

**Background and objectives:**

Home dialysis (peritoneal dialysis and home hemodialysis) is an important renal replacement therapy modality option for patients with end-stage kidney disease. As the Internet has become a primary source for healthcare information, this study aimed to analyze the global and regional interests in home dialysis using Google Trends™ data from January 2004 to March 2024.

**Design, setting, participants, and measurements:**

A comprehensive analysis was conducted using Google Trends™ with the search terms “Peritoneal Dialysis” and “Home Hemodialysis.” This study extracted worldwide trends and detailed regional interests within the United States. Interest levels were quantitatively assessed based on Google Trends™ indices, providing insights into temporal patterns and geographical distributions of public interest.

**Results:**

The study found a fluctuating pattern of global interest in Peritoneal Dialysis, with peak interest in March 2022 and lowest interest in December 2008. The most recent data from March 2024 showed significant interest level of 94, indicating a new upward trend. Mexico exhibited the highest relative interest in Peritoneal Dialysis. Within the United States, Tennessee demonstrated the highest interest. For Home Hemodialysis, the peak interest was in July 2004. The most recent data from March 2024 showed a modest increase in interest. The United States led in highest relative interest for Home Hemodialysis, followed by Australia, Canada, and the United Arab Emirates. Within the United States, Mississippi demonstrated the highest interest.

**Conclusions:**

This study offers crucial insights into the global and regional landscape of interest in home dialysis modalities over time, highlighting the significance of leveraging online platforms to increase public awareness, education, and engagement home dialysis modalities. By understanding the temporal and geographical patterns of interest, healthcare providers, policymakers, and patient advocacy groups can develop targeted strategies to better promote the benefits of home dialysis, address knowledge gaps, and improve access to these life-sustaining treatments.

## Introduction

End-stage kidney disease (ESKD) represents a growing global health challenge, with the prevalence of chronic kidney disease increasing by 29.3% between 1990 and 2017 (1). As the demand for renal replacement therapy (RRT) rises, home-based dialysis modalities, including peritoneal dialysis (PD) and home hemodialysis (HHD), have emerged as viable alternatives to traditional in-center hemodialysis (2). These home-based therapies offer numerous potential benefits, including improved quality of life, greater flexibility, and in some cases, better clinical outcomes and decreased healthcare expenditure (3–5).

Despite these advantages, the uptake of home dialysis modalities remains low and varies significantly across different countries and regions (6). Globally, only approximately 11% of dialysis patients use peritoneal dialysis, with even lower rates of home hemodialysis utilization (7, 8). Understanding the factors that influence these disparities is crucial for developing strategies to increase the adoption of home dialysis where appropriate, especially as the global dialysis population is projected to grow from approximately 3 million in 2017 to 5.4 million by 2030 (9).

In the digital age, the Internet has become a primary source of health information. Online search trends can provide valuable insights into public awareness, interest, and potential concerns regarding healthcare options (10–12). By examining search trends related to peritoneal dialysis and home hemodialysis using tools like Google Trends™ (13), we can gain a deeper understanding of how public interest in these modalities has evolved over time and varies across different geographical areas.

This study aimed to identify temporal trends and geographical variations in public interest in peritoneal dialysis and home hemodialysis using Google Trends™ data from January 2004 to March 2024. This data would address a critical gap in our understanding of public engagement with these life-sustaining therapies. The insights gained from this research may have the potential to inform evidence-based strategies for promoting home dialysis, guide patient education efforts, and ultimately contribute to improving the lives of millions of individuals living with end-stage kidney disease worldwide. This study examining both global trends and detailed regional interests, particularly within the United States, thus offers valuable information for healthcare providers, policymakers, and patient advocacy groups.

## Methods

This observational study utilized Google Trends™ data to analyze global and regional interests in home dialysis modalities from January 2004 to March 2024. Google Trends™ was chosen as the primary data source due to its ability to reflect real-world information-seeking behavior, provide a large sample size, and allow for analysis of trends over extended periods and across various geographical regions.

Google Trends™ (trends.google.com) is a free, publicly available tool that provides data on the relative popularity of search terms over time and across geographical regions. It analyzes a sample of Google search data, eliminating repeated searches from the same person over a short period to avoid skewing the results. The tool has been increasingly used in various fields of research, including healthcare, to gauge public interest and track emerging trends. In the context of our study, Google Trends™ offers a unique opportunity to assess global and regional interest in home dialysis modalities in real-time, potentially reflecting changing awareness, concerns, or information needs of patients, caregivers, and healthcare providers. While it’s important to note that search interest doesn’t directly translate to clinical practice or patient choice, it can provide valuable insights into the public’s engagement with these treatment options and help identify areas for targeted education or policy interventions.

Google Trends Methodology: We used the following specific steps to obtain our data from Google Trends (trends.google.com):

1. Search terms: We used the exact phrases “Peritoneal Dialysis” and “Home Hemodialysis” (including quotation marks to ensure exact matches).
2. Time range: We set the time range from January 1, 2004, to March 31, 2024.
3. Geographic area: For global trends, we selected ‘Worldwide’. For U.S. regional analysis, we selected ‘United States’.
4. Category: We refined our search by selecting the category ‘Health’ to focus on health-related searches.
5. Search type: We used ‘Web Search’ to capture the broadest range of interest.

The study focused on two primary search terms: “Peritoneal Dialysis” and “Home Hemodialysis.” These terms were selected for their specificity to the home dialysis modalities of interest. The 20-year time frame (January 2004 to March 2024) was chosen to capture long-term trends, identify and associate with significant events or policy changes that might have influenced interest levels, and provide a comprehensive view of how interest has evolved with advances in dialysis technologies and healthcare practices.

This study deliberately employed a two-tiered analytical approach, examining trends at both global and regional levels. This dual focus serves several important functions:

1. Comprehensive perspective: The global analysis provides a broad overview of worldwide trends in home dialysis interest, allowing us to identify overarching patterns and compare interest levels across different countries. This global perspective is crucial for understanding the international landscape of home dialysis awareness and potential adoption.
2. Detailed regional insights: The regional analysis, focusing on U.S. states, offers a more granular view of trends within a single country. This level of detail allows us to capture specific variations that might be obscured in the global data, such as state-specific policy impacts, regional healthcare disparities, or localized awareness campaigns.

Google Trends™ provides data in the form of “Interest over time” and “Interest by region” indices, represented on a scale of 0 to 100. Google Trends provides data as relative search volume, where 100 represents peak popularity and all other values are relative to this peak. For example, a value of 50 indicates that the term was half as popular at that time compared to the peak. We conducted temporal analysis to visualize trends and identify peak and trough periods, and analyzed geographical data to rank countries and U.S. states based on their relative interest indices.

## Results

**Figure 1:**
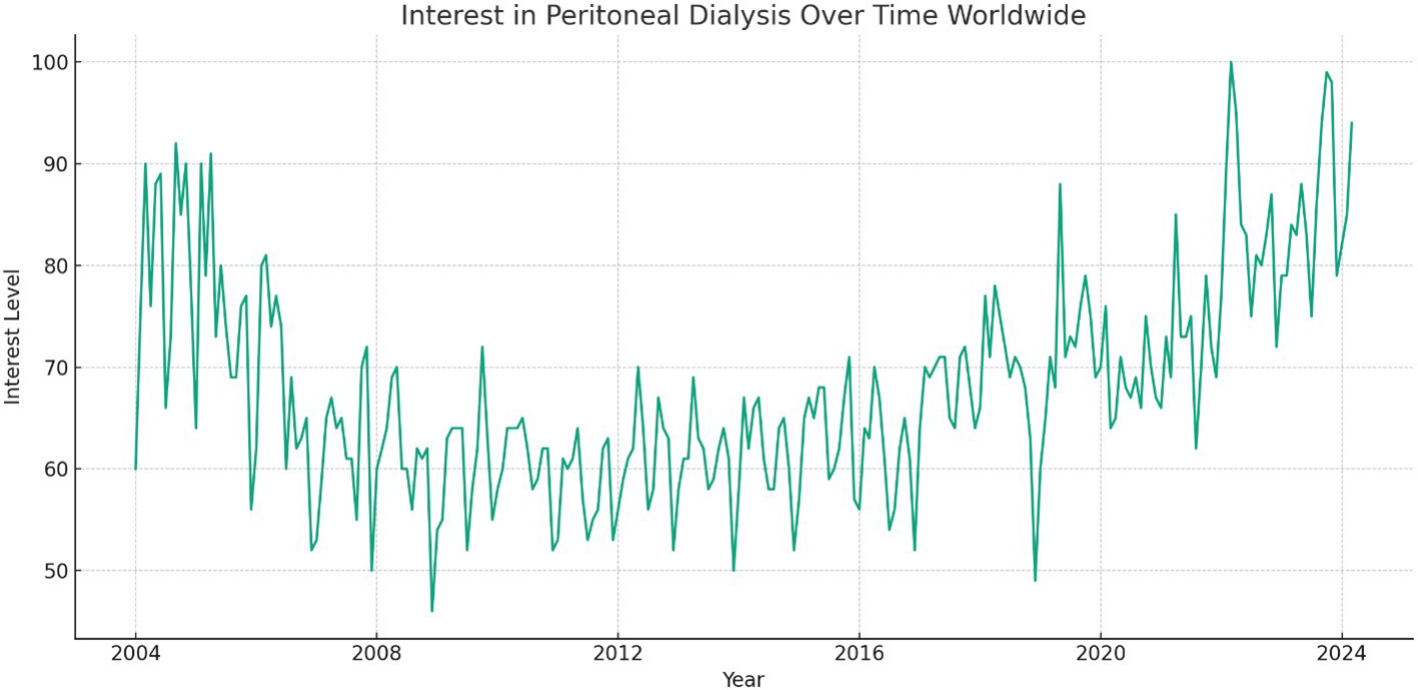
Global Interest in Peritoneal Dialysis from 2004 to 2024.

This study revealed significant fluctuations in global interest for peritoneal dialysis over the 20-year period (**Figure 1**). Interest peaked in March 2022, while the lowest point was observed in December 2008. Notably, the recent data showed a progressive strong upward trend in recent years, reaching a level of 94 most recently.

**Figure 2:**
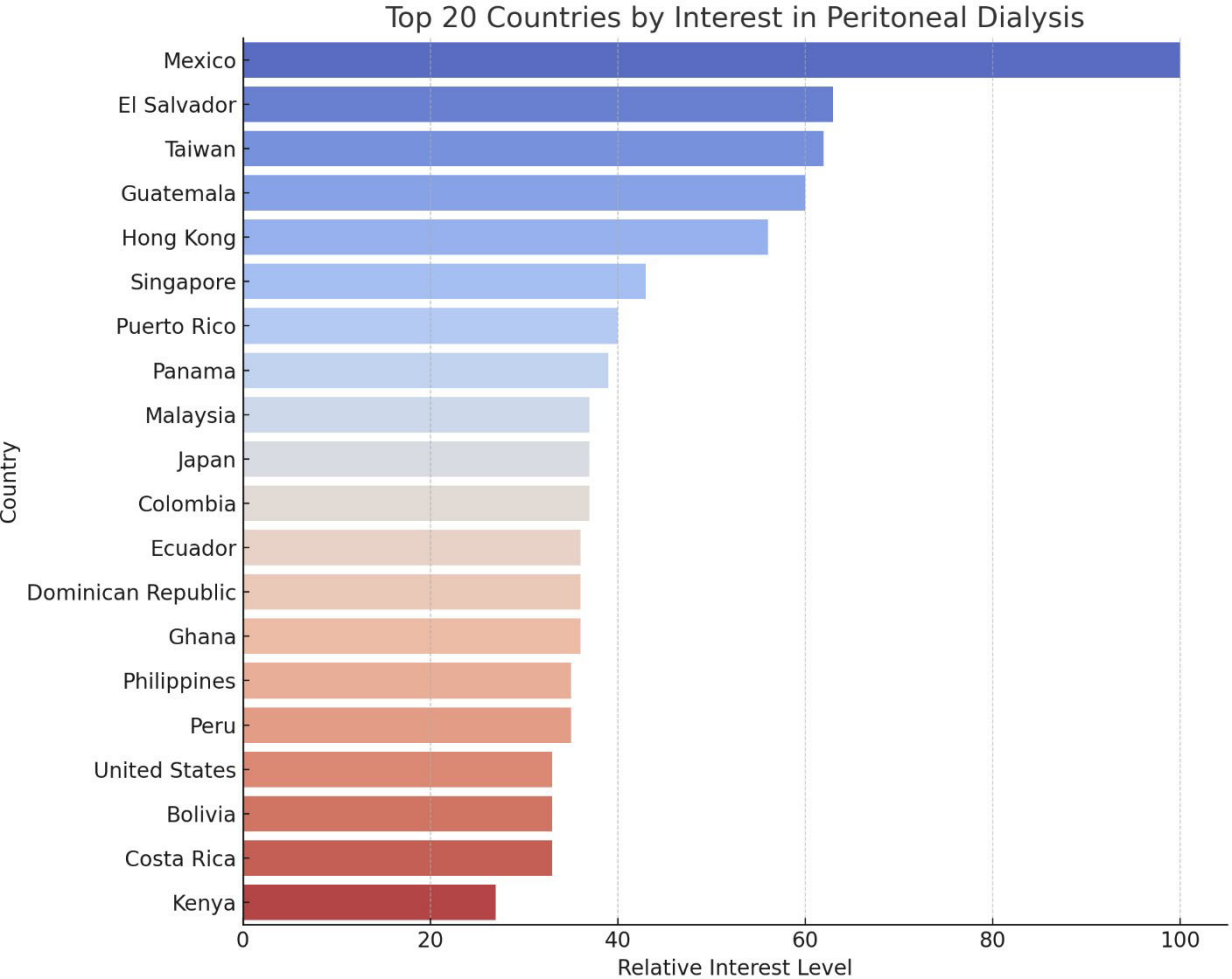
Top 20 Countries by Interest in Peritoneal Dialysis.

On a global scale, Mexico was the country with the highest relative interest in peritoneal dialysis (**Figure 2**). This finding highlights potential global differences in dialysis modality preferences or availability. Within the United States, the state Tennessee demonstrated the highest interest in peritoneal dialysis.

**Figure 3:**
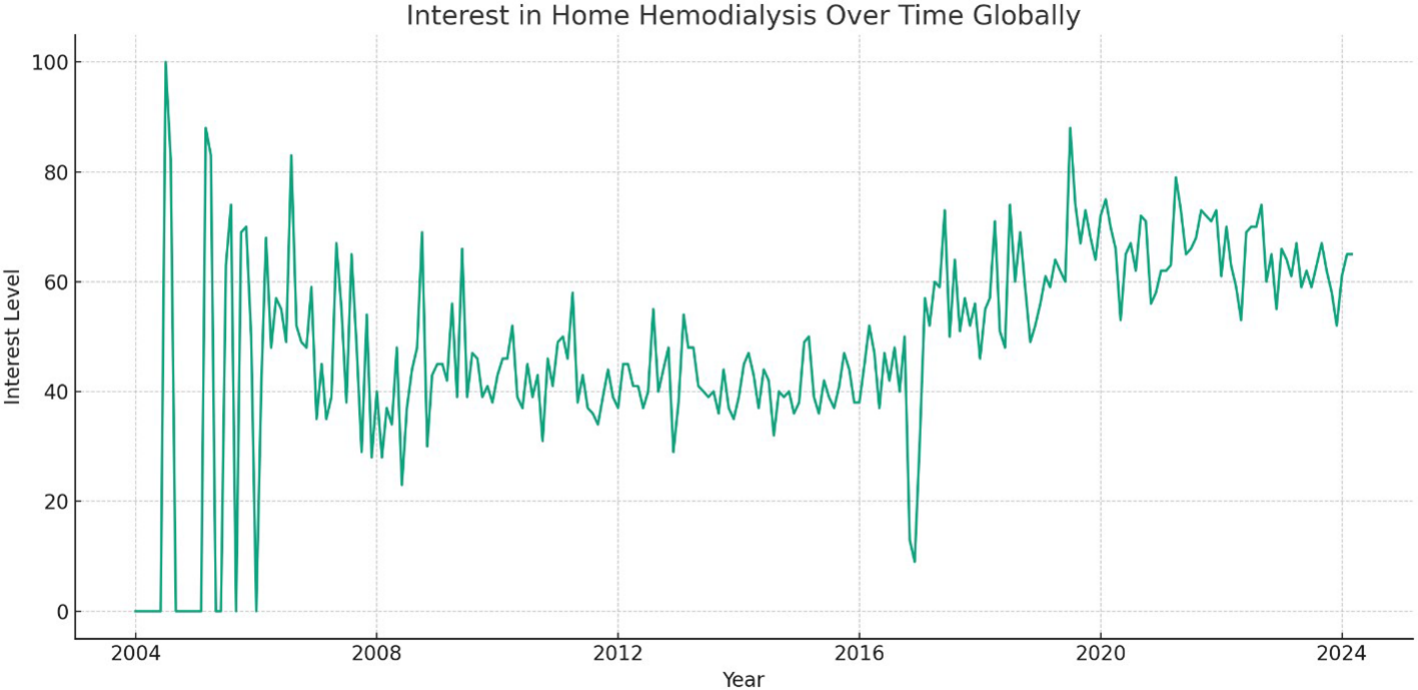
Global Interest in Home Hemodialysis from 2004 to 2024.

The trends for home hemodialysis presented a different pattern compared to peritoneal dialysis. Peak interest in home hemodialysis occurred much earlier in the study period, specifically in July 2004 (**Figure 3**). This early peak could be related to initial enthusiasm or policy changes promoting home hemodialysis at that time. While interest has fluctuated since then, the most recent data from March 2024 indicated a modest increase in interest. This latest uptake suggests a renewed growing attention to home hemodialysis.

**Figure 4:**
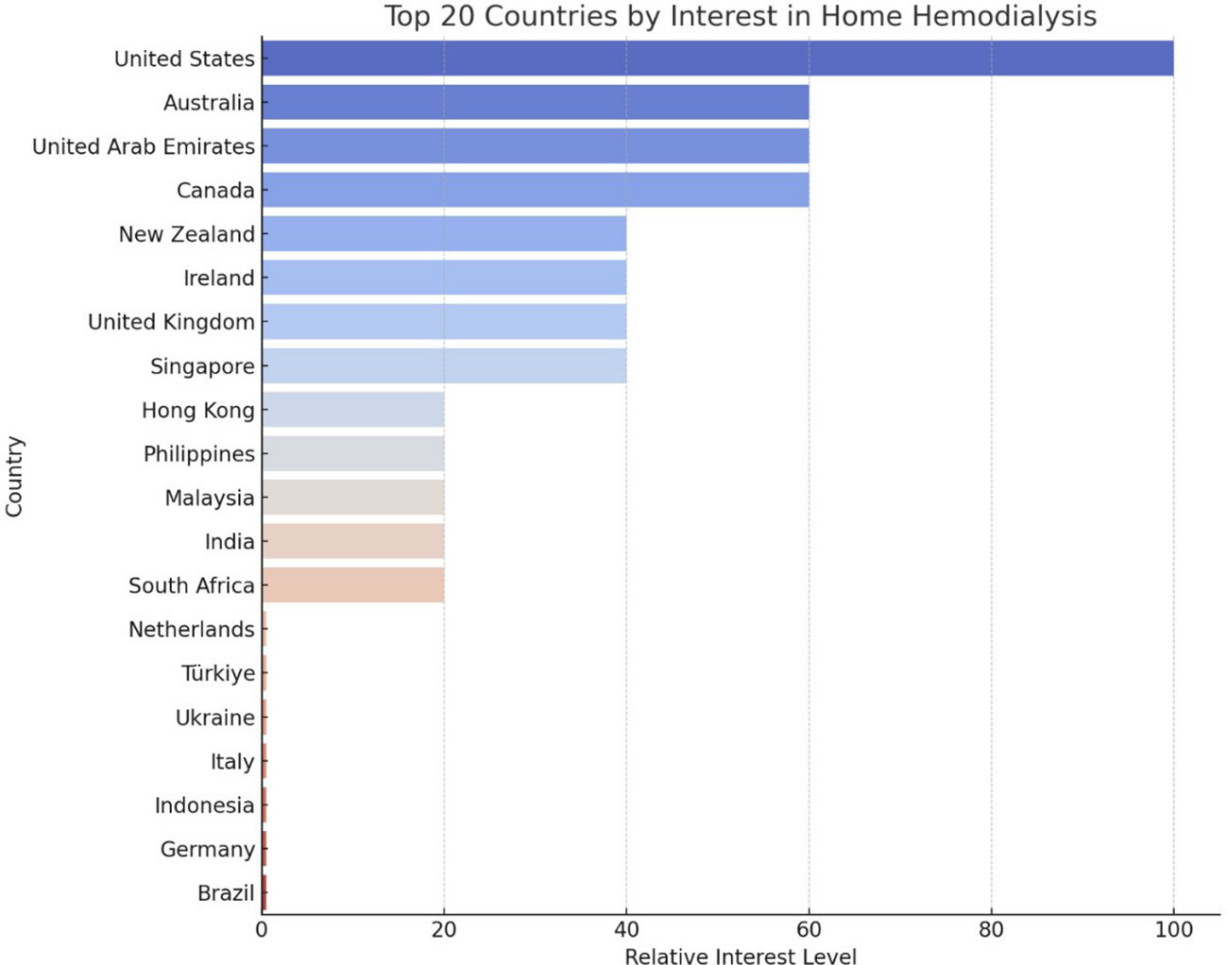
Top 20 Countries by Interest in Home Hemodialysis.

Global interest in home hemodialysis also showed distinct geographical patterns. The United States demonstrated the highest relative interest, followed by Australia, Canada, and the United Arab Emirates (all sharing a relative interest level of 60) (**Figure 4**). New Zealand, Ireland, the United Kingdom, and Singapore followed with a shared relative interest score of 40.

Within the United States, regional variations in home hemodialysis interest were evident. Mississippi led with the highest relative interest score of 100, followed by several states sharing a relative interest score of 77, including Maryland, Tennessee, North Dakota, Alabama, and West Virginia.

## Discussion

This study offers significant insights into the global and regional public interest in home dialysis modalities over a two-decade period from January 2004 to March 2024 by utilizing Google Trends™ data. This analysis identified a notable fluctuation in PD interest. PD interest initially declined but then gradually increased before peaking in March 2022, with subsequently a more recent progressive rise up to the most recent data in March 2024. This data trend was particularly prominent in Mexico and Tennessee. Similarly, HHD showed a peak interest in July 2004, with subsequently more modest fluctuations and rise recently. The highest relative interest was observed in the United States and Mississippi. These findings highlight the dynamic nature of public interest over time and the potential influence of various factors on these trends.

The significant recent increase in interest in PD in March 2024 was unexpected, given the historical fluctuations observed. This surge could be attributed to increased public awareness campaigns, advancements in PD techniques, or changes in healthcare policies promoting home dialysis (14–16). Additionally, the high interest in specific U.S. states such as Tennessee for PD and Mississippi for HHD was surprising, considering that larger states with more comprehensive healthcare systems typically show higher interest. These trends may reflect local healthcare initiatives, the availability of support systems, or demographic factors influencing the preference for home dialysis modalities (17, 18).

The separation of our analysis into global and regional levels revealed important distinctions in home dialysis interest patterns. While global trends provided a broad overview of international interest, the detailed analysis of U.S. states uncovered significant regional variations that might have been overlooked in a solely global analysis. For instance, the high interest in peritoneal dialysis in Tennessee and home hemodialysis in Mississippi highlights the importance of considering local factors in understanding and promoting home dialysis options. This two-tiered approach underscores the need for both international cooperation in promoting home dialysis and tailored, region-specific strategies to address local needs and preferences.

Our analysis revealed a diverse geographical spread of interest in home hemodialysis across different regions of the United States. This widespread interest, not confined to specific areas, potentially reflects varying patient needs, healthcare practices, and the influence of local healthcare policies and educational efforts on home dialysis options. The high interest in states like Mississippi, Maryland, and Tennessee, which represent different geographical and demographic contexts, suggests that factors driving interest in home hemodialysis may be complex and multifaceted. This finding underscores the importance of considering regional variations in healthcare delivery models, patient demographics, and policy environments when developing strategies to promote home dialysis. Furthermore, it highlights the need for tailored approaches in different states to address specific barriers and leverage local factors that may be driving interest in home hemodialysis.

This analysis also revealed fluctuating global interest in PD, with a peak in March 2022 and nadir in December 2008. The most recent data from March 2024 indicated an interest level of 94, suggesting a recent upward trend. Regionally, Mexico exhibited the highest relative interest in PD. Within the United States, Tennessee demonstrated the highest interest. For HHD, the peak interest was recorded in July 2004, with the most recent data from March 2024 showing a modest increase in interest. The United States led in relative interest for HHD, followed by Australia, Canada, and the United Arab Emirates. Within the United States, Mississippi exhibited the highest interest within the United States. Unexpectedly, in the United States, Tennessee and Mississippi showed the highest interest in PD and HHD, respectively, which may reflect local healthcare initiatives or demographic factors influencing these trends. The fluctuations in interest could be attributed to various factors, including policy changes promoting home dialysis, technological advancements, increased awareness due to educational campaigns, and shifts in healthcare delivery models (2, 19–21). It is important to acknowledge that search interest does not equate to adoption or preference for a therapy, highlighting a key limitation of this study.

Previous studies have shown varying levels of interest in home dialysis modalities, but they did not consistently identify the same peak times or geographical interest patterns (22–25). Our study’s findings align with some literature indicating rising awareness and interest in home dialysis, though regional specifics differ (26, 27). The recent surge in interest for PD could be attributed to increased public awareness campaigns (21, 28), advancements in PD techniques (29), a focus on healthcare expenditure, or changes in healthcare policies promoting home dialysis (2). High interest in specific U.S. states may reflect local healthcare initiatives, availability of support systems, or socioeconomic and demographic factors influencing the preference for home dialysis modalities.

Our study, while providing valuable insights, has several limitations. The use of Google Trends data reflects public interest rather than actual adoption rates of home dialysis, potentially influenced by factors like media coverage and internet accessibility. The data represents only those with internet access who use Google as their primary search engine, potentially excluding populations in regions with limited internet penetration. We cannot determine the intent behind searches or whether they represent patient, caregiver, or healthcare professional interests. The relative nature of the data makes it challenging to compare absolute levels of interest across different terms or regions. Major news events, marketing campaigns, or changes in Google’s algorithm could influence search patterns independently of genuine changes in public interest. Additionally, Google Trends does not provide detailed demographic data about users performing searches, limiting our ability to analyze interest patterns across different population subgroups. Regional variations in internet usage may affect result generalizability, potentially underrepresenting areas with limited access. Our focus on specific search terms may not capture all related queries indicating interest in home dialysis modalities. The analysis timeframe, though extensive, may not reflect the most recent trends. Our study design observes trends but cannot establish causal relationships between interest patterns and specific events. While Google Trends’ automatic translation mitigates language barriers, cultural differences in online health information seeking may still influence our findings. Finally, while the findings provide valuable insights into public interest patterns, they may not be entirely representative of global or regional adoption trends. Future research should explore the association between Google Trends interest and actual adoption rates of home dialysis. Additional data sources to capture a more comprehensive picture of public interest could also be incorporated, such as survey data or healthcare utilization statistics, to provide a more comprehensive picture of interest in home dialysis modalities. Once a more comprehensive picture could be created of public interest and home dialysis adoption rates, inferences to significant healthcare events or efforts could be correlated to study their impact. This may allow for healthcare providers and policymakers to more reliably predict and weigh the public impact of future healthcare interventions. These limitations underscore the need for cautious interpretation and further research to complement our findings.

This study underscores the importance of leveraging online platforms to enhance public awareness, education, and engagement in PD and HHD. By understanding temporal and geographical patterns of interest, healthcare providers, policymakers, and patient advocacy groups can develop targeted strategies to promote the benefits of home dialysis, address knowledge gaps, and improve access to these life-sustaining treatments. Continuous monitoring and adaptation of strategies are essential to align with evolving public interest and healthcare needs. Future research should investigate the impact of specific interventions, policies, and educational programs on public interest and adoption of home dialysis, expanding the scope of analysis to include a broader range of search terms and additional data sources. Future studies should investigate the correlation between online interest and actual adoption rates of home dialysis modalities, explore the factors driving high interest in specific regions, and analyze the impact of major events on home dialysis interest. Qualitative studies to understand the motivations behind information-seeking behavior related to home dialysis would also be valuable.

In conclusion, this study provides valuable insights into global and regional interest trends in home dialysis modalities over a 20-year period. While these results offer a novel perspective on public engagement with home dialysis options, they also underscore the need for further research to understand the relationship between online interest and actual therapy adoption. Healthcare providers, policymakers, and patient advocacy groups can use these insights to develop and enhance targeted strategies for promoting home dialysis, addressing knowledge gaps, and improving access to these life-sustaining treatments.

## Data Availability

The original contributions presented in the study are included in the article/supplementary material. Further inquiries can be directed to the corresponding author.
